# Excitatory and inhibitory synaptic dysfunction in mania: an emerging hypothesis from animal model studies

**DOI:** 10.1038/s12276-018-0028-y

**Published:** 2018-04-09

**Authors:** Yeunkum Lee, Yinhua Zhang, Shinhyun Kim, Kihoon Han

**Affiliations:** 1 Department of Neuroscience, College of Medicine, Korea University, Seoul, South Korea; 2 Department of Biomedical Sciences, College of Medicine, Korea University, Seoul, South Korea

## Abstract

Bipolar disorder (BD) is a common psychiatric disorder characterized by recurrent mood swings between depression and mania, and is associated with high treatment costs. The existence of manic episodes is the defining feature of BD, during which period, patients experience extreme elevation in activity, energy, and mood, with changes in sleep patterns that together severely impair their ability to function in daily life. Despite some limitations in recapitulating the complex features of human disease, several rodent models of mania have been generated and characterized, which have provided important insights toward understanding its underlying pathogenic mechanisms. Among the mechanisms, neuronal excitatory and inhibitory (E/I) synaptic dysfunction in some brain regions, including the frontal cortex, hippocampus, and striatum, is an emerging hypothesis explaining mania. In this review, we highlight recent studies of rodent manic models having impairments in the E/I synaptic development and function. We also summarize the molecular and functional changes of E/I synapses by some mood stabilizers that may contribute to the therapeutic efficacy of drugs. Furthermore, we discuss potential future directions in the study of this emerging hypothesis to better connect the outcomes of basic research to the treatment of patients with this devastating mental illness.

## Bipolar disorder: a nervous connection

Studies in rodents offer insights into bipolar disorder that may help understanding and treatment of this common and debilitating condition. Kihoon Han and colleagues at Korea University in Seoul review research using mice and rats to model the episodes of mania in patients with bipolar disorder. The research supports an emerging hypothesis implicating specific problems with nervous transmission in the brain in the onset of mania. The hypothesis suggests that the transmission of signals between particular nerve cells whose normal function is either to excite or to inhibit other nerve cells may be involved. It also indicates regions of the brain most involved in manic episodes. Changes at the affected nerve junctions—called synapses—brought about by mood-stabilizing drugs are examined. The hypothesis suggests new approaches to treatment options for researchers to explore.

## Introduction

Bipolar disorder (BD) is a common and devastating mental illness, characterized by recurrent mood swings between depression and mania with intervening euthymic states^1^. BD affects approximately 1–2.5% of the world’s population^2^, and the World Health Organization recognizes BD as the sixth leading cause of disability. Existence of manic episodes is the defining feature of BD, which differentiates it from unipolar major depressive disorder. The symptoms of manic episode include hyperactivity, impulsivity, elevated mood, inflated self-esteem, reduced anxiety, decreased need for sleep, and sometimes psychosis^1^. Both environmental and genetic risk factors contribute to the pathogenesis of mania, but the detailed molecular and cellular pathways underlying mania remain largely unknown.

So far, several rodent models of mania have been generated and characterized. Traditionally, pharmacological (e.g., psychostimulant amphetamine-induced) and environmental (e.g., sleep deprivation-induced) models were studied, but more recently various genetic models (i.e., knockout (KO), knock-in (KI), and overexpressing transgenic (TG) mice) have been developed^3^. Even with some limitations in satisfying all three (construct, face, and predictive) validities as a disease model^3, 4^, each of these rodent models has provided important insights toward understanding the pathogenic mechanisms of mania. For example, manic-like behaviors of rodents injected with amphetamine or those expressing lower levels of dopamine transporter^3^, together with clinical evidence of higher dopamine levels during manic episodes^5^, supported hyperdopaminergic activities as a major pathophysiology of mania. Nevertheless, the clinical heterogeneity of mania, such as the differential response to certain pharmacological treatments^6^, suggests the possibility that other pathogenic mechanisms can still exist.

Neuronal excitability is tightly controlled by excitatory and inhibitory (E/I) synaptic balance, and dysfunction of this process has been strongly associated with numerous neurodevelopmental and neuropsychiatric disorders, including autism spectrum disorders (ASDs), intellectual disability (ID), and schizophrenia (SCZ)^7–10^ This could involve various underlying mechanisms ranging from abnormal expression and function of pre- or postsynaptic molecules^11^ to impaired maturation of certain neuronal cell types, such as γ-aminobutyric acid (GABA)ergic inhibitory interneurons^12^. Despite some evidence suggesting abnormal GABAergic interneurons in BD^13^, E/I synaptic dysfunction in mania has been relatively unexplored compared to that in other brain disorders. In the current review, we highlight recent studies of rodent manic models with impairments in E/I synaptic development and function. We also summarize thus far identified molecular and functional changes of E/I synapses by some mood stabilizers. Lastly, we discuss current limitations and potential future directions of this emerging hypothesis to better connect the outcomes of basic research to the treatment of patients with BD. For more general and comprehensive coverage of animal models of mania, we refer to recent excellent reviews^3,14^.

## Animal models of mania with E/I synaptic dysfunction

### *Shank3*-overexpressing TG mice

The SH3 and multiple ankyrin repeat domains 3 (*SHANK3*, also called *PROSAP2*) gene encodes a core scaffold protein in postsynaptic density (PSD) of the excitatory synapse^15^. By interacting with hundreds of different molecules in PSD^16,17^, including membrane proteins, signaling molecules, and cytoskeletal components, Shank3 organizes the macromolecular protein complex and is critically involved in proper development and function of excitatory synapses. Clinically, deletions and duplications of the chromosomal region (22q13) containing *SHANK3* and various point mutations of *SHANK3* have been identified in patients with ASDs, ID, SCZ, BD, and attention deficit hyperactivity disorder (ADHD)^18,19^. Han et al.^16^ recently identified two patients with small 22q13 duplications that likely include only *SHANK3*, and found that these patients were diagnosed with hyperkinetic disorders, BD, and ADHD, respectively. Notably, the symptoms of the patient with ADHD were not improved by treatment with amphetamine, a common medication for ADHD, suggesting the possibility that the disorder could not be typical ADHD, but more likely BD. To model *SHANK3* duplications, Han et al. generated *Shank3* TG mice that mildly overexpress Shank3 proteins (to approximately 150%) compared to wild-type (WT) mice. Indeed, the *Shank3* TG mice displayed several manic-like behaviors, including locomotor hyperactivity and hypersensitivity to amphetamine in the open-field test (OFT), reduced despair-like behavior in the tail-suspension test (TST), increased acoustic startle response, reduced prepulse inhibition (PPI), and abnormal circadian rhythms, some of which responded to valproate (VPA), a Food and Drug Administration (FDA)-approved mood stabilizer and anticonvulsant for the treatment of manic or mixed episodes in BD^6,16^.

The E/I synaptic morphology and function of *Shank3* TG mice were characterized mainly in the hippocampus^16^. The number of excitatory synapses was increased, while that of inhibitory synapses was decreased in the cultured hippocampal neurons of *Shank3* TG mice compared to WT neurons. Functionally, amplitude, but not frequency, of spontaneous excitatory postsynaptic currents (sEPSCs) was significantly increased, while frequency, but not amplitude, of miniature inhibitory postsynaptic currents (mIPSCs) was decreased in the CA1 pyramidal neurons of acute hippocampal slices from *Shank3* TG mice. Consistent with the shifted E/I synaptic balance toward more excitation but less inhibition, the *Shank3* TG mice displayed abnormal electroencephalography (EEG) patterns in the frontal cortex and hippocampus, and exhibited spontaneous seizures. The abnormal EEG was also rescued by VPA treatment. At the molecular level, Han et al. found that Shank3 interacted with various actin-regulatory proteins to promote actin polymerization in the excitatory postsynaptic sites, thereby increasing the number of dendritic spines in the CA1 pyramidal neurons of *Shank3* TG mice^16,20^.

It was unexpected that increased expression of Shank3 proteins that exclusively localize to excitatory, but not inhibitory, postsynaptic sites caused reduced number and function of inhibitory synapses in the *Shank3* TG hippocampus. One possible explanation would be impaired maturation and/or function of GABAergic inhibitory neurons. However, Lee et al. recently showed that the densities of parvalbumin (PV)- and somatostatin-positive interneurons were normal in the hippocampus, striatum, and medial prefrontal cortex (mPFC) of *Shank3* TG mice^21^. Therefore, it is more likely that the inhibitory synaptic changes observed in *Shank3* TG mice could be due to the cell-autonomous postsynaptic changes of principal neurons that may involve a shift in distribution of certain actin-regulatory proteins, such as Mena and Profilin2, from inhibitory to excitatory postsynaptic sites^16^.

Despite the abnormal EEG in the frontal cortex of *Shank3* TG mice, it remains to be directly investigated whether the E/I synaptic morphology and function are also altered in other brain regions, such as the striatum and mPFC, of the mice. In this regard, it is notable that in the several lines of *Shank3* KO and KI mice modeling ASDs and SCZ, changes in the number and function of E/I synapses in these brain regions have been observed^22–24^. Moreover, Lee et al.^25^ recently showed increased levels of actin filaments (F-actin) in the striatum of *Shank3* TG mice, similar to the hippocampus. The detailed mechanism of how VPA treatment rescued the manic-like behaviors of *Shank3* TG mice is also unknown. One possibility is that VPA enhanced the GABAergic inhibitory synaptic transmission of *Shank3* TG mice by inhibiting a GABA-catabolizing enzyme, GABA transaminase, and thereby normalizing the E/I synaptic balance^26^.

### Forebrain-specific *Plcg1* KO mice

Phospholipase Cγ1 (PLCγ1) is an enzyme that, when activated by receptor tyrosine kinases, hydrolyzes membrane-bound phosphatidylinositol 4,5-bisphosphate (PIP_2_) to generate the second messengers diacylglycerol and inositol-1,4,5-triphosphate (IP_3_)^27^. In neurons, brain-derived neurotrophic factor (BDNF), through its receptor tropomyosin receptor kinase B (TrkB), activates PLCγ1 together with other downstream signaling components, including Ca^2+^/calmodulin-dependent protein kinase II (CaMKII), extracellular signal-regulated kinase, and cAMP response element-binding protein to regulate synaptic development, function, and plasticity^28,29^. Despite some studies suggesting the association between *PLCG1* polymorphism and BD^30,31^, the functional roles of PLCγ1 in mature neurons in vivo has not been investigated because of the lethality of conventional *Plcg1* KO mice at an early embryonic stage. To solve this problem, recently, Yang et al.^32^ generated forebrain-specific *Plcg1* KO mice by crossing the floxed *Plcg1* mice with *CaMKII-Cre* mice (*Plcg1*^*f/f*^*; CaMKII* mice). The *Plcg1*^*f/f*^*; CaMKII* mice exhibited several manic-like behaviors, including locomotor hyperactivity in the OFT, reduced anxiety-like behavior in the elevated plus maze (EPM) test, reduced despair-like behavior in the forced swim test (FST), hyperhedonic behavior in the sucrose preference test (SPT), and increased acoustic startle response, many of which were normalized by treatment with VPA or lithium, another FDA-approved mood stabilizer for the treatment of mania in BD^33^. In addition to the manic-like behaviors, the mice showed impaired learning and memory in the auditory and contextual fear conditioning tests.

The basal excitatory synaptic transmission of *Plcg1*^*f/f*^*; CaMKII* mice was normal in the hippocampal Schaffer collateral (SC)-CA1 synapses as measured by the input-output relationship and the *N*-methyl-d-aspartic acid (NMDA) receptor to α-amino-3-hydroxy-5-methyl-4-isoxazole propionate (AMPA) receptor current ratio (NMDAR/AMPAR ratio)^32^. Moreover, amplitude and frequency of miniature EPSCs (mEPSCs) were normal in the CA1 pyramidal neurons of *Plcg1*^*f/f*^*; CaMKII* mice. Consistently, expression levels of several excitatory synaptic proteins and number of dendritic spines in the hippocampus of *Plcg1*^*f/f*^*; CaMKII* mice were comparable to those of WT mice. In contrast, the *Plcg1*^*f/f*^*; CaMKII* mice showed defects in the inhibitory synapses of the hippocampus and dorsal striatum^32^. Specifically, frequency, but not amplitude, of mIPSCs in the CA1 pyramidal neurons was reduced in the hippocampal slices of *Plcg1*^*f/f*^*; CaMKII* mice compared to WT mice. Notably, in the dorsal striatum, where D1 (dopamine receptor D1-expressing)- and D2 (dopamine receptor D2-expressing)-type medium spiny neurons (MSNs) account for the majority (>90%) of the neuronal population^34^, the amplitude of mIPSCs was reduced only in the D1 type, but not D2 type, MSN of *Plcg1*^*f/f*^*; CaMKII* mice. This is somewhat consistent with the behavioral phenotype of the mice, as activation of D1-type MSN promotes locomotor activities^35^.

These functional defects of inhibitory synapses were explained by a reduced number of GABAergic inhibitory presynaptic terminals on the principal neurons^32^. At the molecular level, both basal and BDNF-induced phosphorylation of CaMKIIα were significantly decreased at the inhibitory postsynaptic sites of *Plcg1*^*f/f*^*; Nestin* cultured hippocampal neurons^32^. Furthermore, the surface expression of inhibitory GABA_A_ receptor α1 subunit, which is known to be regulated through its phosphorylation by CaMKIIα, was also decreased in the *Plcg1*^*f/f*^*; Nestin* hippocampal neurons compared to WT neurons.

In addition to the E/I synaptic dysfunction in the hippocampus and striatum, BDNF-TrkB-dependent long-term potentiation, a type of synaptic plasticity, at the SC-CA1 synapses of *Plcg1*^*f/f*^*; CaMKII* hippocampus was also defective, which could explain the impaired learning and memory of the mice. Together, these findings suggest that BDNF-TrkB-PLCγ1 signaling is required for the proper development and function of inhibitory synapses and for the normal synaptic plasticity in the striatum and hippocampus, defects of which contribute to manic-like behaviors and impaired learning and memory in the forebrain-specific *Plcg1* KO mice.

### *Ank3-1b* heterozygous mice

The *ANKYRIN 3* (*ANK3*) gene encodes multiple isoforms of Ankyrin-G proteins that link membrane proteins to the β-spectrin/actin cytoskeleton and thereby organizing macromolecular complexes at the specialized plasma membrane compartments^36^. In neurons, Ankyrin-G proteins are involved in the formation and maintenance of axon initial segments (AISs) and node of Ranvier, membrane compartments critical for action potential generation and propagation, where they connect several ion channels to the cytoskeleton^37^. Notably, the *ANK3* gene has been repeatedly associated with BD from several large-scale genome-wide association studies^38–40^.

Recently, Leussis et al.^41^ generated two different mouse models with reduced *Ank3* expression and found that both showed some manic-like behaviors. In the first model, *Ank3* expression was specifically reduced in the hippocampal dentate gyrus (DG) using the lentivirus expressing short hairpin RNAs targeting *Ank3*. These mice showed reduced anxiety-like behavior in multiple behavioral assays, including the EPM, light-dark transition, and novelty-suppressed feeding tasks. In addition, the mice showed increased home-cage activity during the light, but not the dark, phase. Both reduced anxiety-like behavior and increased activity were rescued by treatment with lithium^41^. The second model was generated by disrupting the exon 1b locus of *Ank3* (*Ank3-1b*), which resulted in decrease of the Ankyrin-G isoforms containing exon 1b in several brain regions, including the DG, cortex, and cerebellum^41,42^. Because of the progressive early-onset ataxia in homozygous *Ank3-1b*^*−/−*^ mice^42^, the behavioral phenotypes of heterozygous *Ank3-1b*^*+/−*^ mice were characterized and compared to those of WT mice. Similar to the mice with DG-specific decrease of *Ank3* expression, *Ank3-1b*^*+/−*^ mice displayed reduced anxiety-like behavior in the EPM, light-dark transition, and novelty-suppressed feeding tasks. Moreover, *Ank3-1b*^*+/−*^ mice exhibited hyperhedonic behavior in the SPT. Intriguingly, after chronic social isolation stress, the manic-like behaviors of *Ank3-1b*^*+/−*^ mice were shifted to depression-like behaviors^41^. Specifically, singly housed *Ank3-1b*^*+/−*^ mice showed increased anxiety-like behavior in the EPM test, anhedonic behavior in the SPT, and despair-like behavior in the FST, compared to singly housed WT mice. This elevated susceptibility to stress of *Ank3-1b*^*+/−*^ mice was partly explained by the higher plasma levels of corticosterone compared to WT mice, both under basal and acute stress conditions^41^.

Alternative first exons (1a/1a′, 1e, and 1b) of *ANK3* gene encode distinct N-terminal peptide sequences of Ankyrin-G isoforms^43,44^. Importantly, Lopez et al.^44^ revealed that PV-positive GABAergic inhibitory interneurons express only Ankyrin-G isoforms containing exon 1b, while excitatory principal neurons express the isoforms containing either exon 1e alone, or both 1e and 1b. Consistently, clustering of voltage-gated sodium channels at the AISs of PV-positive interneurons was significantly decreased in *Ank3-1b*^*+/−*^ mice compared to WT mice. Further supporting the interneuron dysfunction, *Ank3-1b*^*+/−*^ and *Ank3-1b*^*−/−*^ mice exhibited abnormal EEG and seizures in an *Ank3* gene dose-dependent manner (i.e., they were more severe in homozygous *Ank3-1b*^*−/−*^ than in heterozygous *Ank3-1b*^*+/−*^ mice). In addition, the detailed electrophysiological analysis showed reduced intrinsic excitability of the PV-positive interneurons in *Ank3-1b*^*−/−*^ mice compared to WT mice^44^. Combining the abovementioned studies, it is conceivable that functional changes of PV-positive GABAergic inhibitory neurons and their synapses in some brain regions of *Ank3-1b*^*+/−*^ mice could contribute to manic-like behaviors.

More recently, Zhu et al.^45^ generated and characterized a mouse model with conditional disruption of *Ank3* in pyramidal neurons of adult forebrain (*Ank3*^*f/f*^*; CaMKII*). All three major isoforms of Ankyrin-G proteins (190, 270, and 480 kDa) were decreased in the forebrain regions of *Ank3*^*f/f*^*; CaMKII* mice. These mice, similar to *Ank3-1b*^*+/−*^ mice, displayed several manic-like behaviors responsive to lithium and VPA, which could be shifted to depression-like behaviors after repeated social defeat stress. There was a loss of sodium and potassium channels at AISs of pyramidal neurons in *Ank3*^*f/f*^*; CaMKII* mice. Moreover, the number of inhibitory synapses innervating pyramidal neuron AISs was significantly decreased in the cortex of *Ank3*^*f/f*^*; CaMKII* mice. Consistent with disinhibition, c-fos expression was increased in cortical pyramidal neurons of the mice, indicating increased cortical activity^45^. Therefore, both *Ank3-1b*^*+/−*^ and *Ank3*^*f/f*^*; CaMKII* mice have some defects in the inhibitory synaptic function. Nevertheless, in addition to the AISs, Ankyrin-G proteins also localize to the dendritic spines of glutamatergic excitatory postsynapse where they regulate dendritic spine morphology, and excitatory synaptic transmission and plasticity^46^. Therefore, morphological and functional changes of excitatory synapses in some brain regions of *Ank3-1b*^*+/−*^ and *Ank3*^*f/f*^*; CaMKII* mice cannot be excluded, details of which remain to be investigated.

### *ClockΔ19* mutant mice

Abnormalities in circadian rhythms and sleep disturbance have been linked with BD and manic episodes^47,48^. The circadian locomotor output cycles kaput (*CLOCK*) gene encodes a CLOCK protein that forms a heterodimer with brain muscle ARNT-like 1 (BMAL 1) and functions as a critical transcriptional regulator in the feedback network of the molecular clock^49^. Notably, polymorphisms of *CLOCK* and *BMAL 1* have been associated with BD^50,51^. The *ClockΔ19* mutant mice have *N*-ethyl-*N*-nitrosourea-induced single base mutation at the 5′ splice donor site of intron 19, which results in loss of exon 19 during splicing and thereby produces a dominant negative CLOCK protein^52^. Indeed, *ClockΔ19* mutant mice show significant circadian rhythm defects both in the molecular and behavioral levels^53^.

McClung’s group has reported a series of studies demonstrating the manic-like behaviors of *ClockΔ19* mutant mice and revealing the underlying molecular and neural circuit mechanisms. *ClockΔ19* mutant mice exhibited locomotor hyperactivity both in novel and familiar environments, increased response to various reward stimuli, hyperhedonic behavior in the SPT, reduced despair-like behavior in the FST, and reduced anxiety-like behavior in the EPM test, some of which were rescued by treatment with lithium^54–56^. Intriguingly, the manic-like behaviors of *ClockΔ19* mutant mice were time-dependent; the behaviors were significant during the day time, but were normalized to WT levels (euthymic states) during the night time^57^. Hyperdopaminergic activity due to the increased firing rate of dopaminergic neurons in the ventral tegmental area (VTA) was shown as a key neuronal mechanism for the manic-like behaviors of *ClockΔ19* mutant mice^55^. Specifically, viral expression of functional CLOCK proteins in the VTA of mutant mice was sufficient to rescue the manic-like behaviors^54^. Moreover, the dopaminergic neuron firing rate, tyrosine hydroxylase expression, and dopamine synthesis in the VTA of mutant mice coincided with the time-dependent behavioral changes^57^.

The nucleus accumbens (NAc) is a brain region of the ventral striatum critically involved in mood, reward, and addiction-related behaviors, and the MSNs of NAc get dopaminergic inputs from the VTA and excitatory glutamatergic inputs from the prefrontal cortex^58^. The hyperdopaminergic activity of *ClockΔ19* mutant mice could lead to the circuit level changes of NAc and thereby contribute to the manic-like behaviors. Indeed, Dzirasa et al.^59^ revealed neurophysiological abnormalities of the NAc microcircuits in *ClockΔ19* mutant mice by performing simultaneous in vivo extracellular recordings from the VTA, NAc, and prefrontal cortex. Specifically, low-gamma oscillations and single neuron phase coupling were defective in the NAc of *ClockΔ19* mutant mice, which were normalized by treatment with lithium. At the molecular level, total-, phospho (S845)-, and surface expression levels of the GluA1, but not the GluA2, subunit of AMPA-type glutamate receptors were decreased in the NAc of mutant mice compared to WT mice, suggesting that increased dopamine release may indirectly lower the excitatory synaptic transmission of NAc in *ClockΔ19* mutant mice^3,59,60^. Consistent with the biochemical changes, mEPSC amplitude, but not frequency, and AMPAR/NMDAR ratio were decreased in the MSNs of *ClockΔ19* mutant NAc^60^. Furthermore, viral-mediated overexpression of the GluA1 subunit of the AMPA receptor in the NAc was sufficient to normalize some manic-like behaviors of the mutant mice, including reduced anxiety-like behavior in the EPM test and increased reward sensitivity in the conditioned place preference test^60^.

Together, reduced excitatory synaptic function of the MSNs in NAc, potentially as a consequence of increased activity of dopaminergic inputs from the VTA, could be an important mechanism for the manic-like behaviors of *ClockΔ19* mutant mice. It remains to be investigated whether the inhibitory synapses of MSNs in *ClockΔ19* mutant mice have also any molecular and functional changes. Moreover, whether the E/I synapses of D1- and D2-type MSNs could be differentially altered in the mutant mice is an interesting topic for future research^61^.

### Sleep-deprived animals

Clinically, reduced sleep or sleep disturbances can trigger and worsen manic episodes^62^. Similarly, sleep deprivation protocols, usually obligating rodents to remain awake on a small platform surrounded by water for an extended period of time (72 h), have long been used to generate manic models^63^. The sleep-deprived animals indeed exhibit several manic-like behaviors, including locomotor hyperactivity, aggressive behavior, hypersexuality, and increased stereotypy, which could be normalized by treatment with lithium^63,64^.

A few studies have suggested that protein kinase C (PKC) could mediate important molecular mechanisms underlying the manic-like behaviors of sleep-deprived animals. The levels of PKC activity and phosphorylation of some PKC substrates were increased in the frontal cortex of sleep-deprived rats^65^. Moreover, treatment of quercetin, a PKC inhibitor, prevented sleep deprivation-induced locomotor hyperactivity in mice^66^. Among the increased PKC-dependent phosphorylation by sleep deprivation, those on the S896 of the GluN1 subunit of the NMDA receptor and the T840 of the GluA1 subunit of the AMPA receptor are notable^65^. S896 phosphorylation of GluN1 is involved in regulating intracellular trafficking and surface expression of NMDA receptors^67^. T840 phosphorylation of GluA1 could enhance channel conductance of AMPA receptors^68^. Therefore, it is possible that sleep deprivation could affect excitatory synaptic function and plasticity in the frontal cortex, which then contribute to manic-like behaviors.

Sleep deprivation-induced alterations in synaptic function and plasticity have been extensively investigated in different brain regions of rodents, although, in many of the studies, behavioral changes after various sleep deprivation protocols were not characterized, and therefore the synaptic changes may not be directly associated with mania. In the CA1 and DG neurons of the hippocampus, sleep deprivation resulted in impairment of long-term potentiation^69,70^. Consistent with the notion that NMDA receptors are critical upstream regulators of synaptic plasticity^71^, surface expression of NMDA receptors and NMDA receptor-mediated currents were reduced after sleep deprivation^72,73^. Meanwhile, AMPA receptor function, as measured by mEPSCs, was normal in the hippocampus^73^. Impaired hippocampal long-term potentiation, nevertheless, could be more associated with learning and memory deficits after sleep deprivation than with manic-like behaviors^70^. In the frontal cortex, mild sleep deprivation increased mEPSC amplitude and frequency of layer II/III pyramidal neurons^74^. In the deep layers (V/VI) of the mPFC, sleep deprivation decreased amplitude, but not frequency, of mEPSCs without affecting the properties of mIPSCs^75^. Together, sleep deprivation could result in diverse changes of E/I synaptic function, depending on the brain regions and sleep deprivation protocols. Therefore, more investigations are necessary to understand the causal relationship between E/I synaptic changes of certain brain regions and manic-like behaviors after sleep deprivation.

## Molecular and functional changes of E/I synapses by mood stabilizers

Clinically, antipsychotics (such as haloperidol, risperidone, and olanzapine) and mood stabilizers (such as lithium, VPA, lamotrigine, and carbamazepine) are most commonly used for the acute and long-term management of mania, respectively^1,6^. Antipsychotics exhibit high-affinity antagonism toward dopamine and serotonin receptors, but their chronic treatment could also affect glutamate receptor expression^76^. The molecular targets and mechanisms of action for mood stabilizers are more complex than those for antipsychotics, and there are still many questions remaining in the field^77,78^. For example, lithium alone has multiple direct targets, including inositol monophosphatase, phosphoglucomutase, and glycogen synthase kinase-3, and additionally affects diverse downstream pathways indirectly^26,79^.

In Table 1, we summarize animal model and in vitro-cultured neuron studies showing E/I synaptic changes by mood stabilizers, at the molecular, cellular, and functional levels. It is notable that possibly depending on the treatment and measurement conditions, synaptic changes in a certain brain region by a single mood stabilizer can be diverse, or even opposite. Therefore, further investigations are necessary to understand whether and to what extent each of these synaptic changes contributes to the therapeutic efficacy of drugs.

**Table 1:** Summary of the E/I synaptic changes by mood stabilizers

Mood stabilizer	E/I synaptic changes	Species and brain regions	References
Lithium	**Molecular**		
	Increases synaptic expression of AMPAR GluA2 subunit	Mouse HIP and rat cultured HIP neurons	^89^
	Increases synaptic clustering of gephyrin	Rat cultured HIP neurons	^90^
	Increases synaptic expression of GABA_B_R	Rat frontal CTX	^91^
	Decreases mRNA levels of *Homer1b/c* and *Shank1*	Rat CTX and STR	^92^
	Decreases surface expression of AMPAR GluA1 subunit	Mouse cultured HIP neurons	^93^
	Decreases tyrosine phosphorylation of NMDAR NR2A subunit	Rat HIP	^94^
	Decreases tyrosine phosphorylation of NMDAR NR2B subunit	Rat cultured CTX neurons	^95^
	Decreases synaptosomal and surface expression of AMPAR GluA1 and GluA2 subunits	Rat HIP and cultured HIP neurons	^96–98^
	**Morphological**		
	Increases number of excitatory synapses	Rat cultured HIP neurons	^99^
	Increases number of dendritic spines in *Dixdc1* KO mice	Mouse primary somatosensory CTX	^100^
	Decreases number of dendritic spines in *fmr1* KO mice	Mouse mPFC	^101^
	**Functional**		
	Increases AMPAR opening probability	Rat HIP	^102^
	Increases input-output relationship and long-term potentiation	Rat DG	^103^
	Increases excitatory presynaptic transmission	Rat HIP	^104, 105^
	Decreases AMPAR/NMDAR ratio	Rat HIP	^97^
	Decreases amplitude of AMPAR-mediated mEPSC	Mouse cultured HIP neurons	^93^
	Decreases long-term depression	Rat HIP	^106^
Valproate	**Molecular**		
	Increases level of GABA and activity of GAD	Mouse whole brain	^107^
	Increases GABA but decreases glutamate levels	Mouse whole brain	^108^
	Increases levels of glutamate transporters and capacity of glutamate uptake	Rat HIP	^109^
	Decreases mRNA levels of *Homer1b/c* and *Shank1*	Rat CTX and STR	^92^
	Decreases synaptosomal and surface expression of AMPAR GluA1 and GluA2 subunits	Rat HIP and cultured HIP neurons	^96–98^
	**Morphological**		
	Increases number of dendritic spines in prenatal VPA-induced ASD model mice	Mouse HIP	^110^
	**Functional**		
	Increases GABA-induced inhibition in single unit recording	Rat CTX	^111^
	Decreases amplitude of NMDAR-mediated EPSP	Rat AMYG	^112^
	Decreases NMDAR-mediated EPSP slope but increases GABAR-mediated IPSP slope	Rat HIP	^113^
	Decreases amplitude of non-NMDAR-mediated EPSP	Rat HIP	^114^
Lamotrigine	**Molecular**		
	Increases surface expression of AMPAR GluA1 and GluA2 subunits	Rat cultured HIP neurons	^98^
	Increases level of GABA and activities of GAD and GABA transaminase	Rat HIP	^115^
	**Functional**		
	Decreases presynaptic glutamate release	Rat HIP and AMYG	^116–118^
	Decreases presynaptic glutamate release and postsynaptic AMPAR-mediated current	Rat DG	^119^
	Decreases presynaptic glutamate release but increases presynaptic GABA release	Rat entorhinal CTX	^120^
	Decreases GABA_A_R-mediated synaptic transmission	Rat AMYG	^121^
Carbamazepine	**Molecular**		
	Increases synaptic expression of GABA_B_R	Rat frontal CTX	^91^
	Increases GABA but decreases glutamate levels	Rat prefrontal CTX and thalamus	^122^
	**Functional**		
	Increases excitatory, but not inhibitory, synaptic transmission	Mouse HIP	^123^
	Increases GABA-induced current of GABA_A_R	Rat cultured CTX neurons	^124^
	Decreases presynaptic glutamate release	Rat HIP	^116, 117, 125^
	Decreases excitatory synaptic transmission and postsynaptic response of glutamate receptors	Rat HIP	^126^

*AMYG* amygdala, *CTX* cortex, *Dixdc1* DIX domain containing-1, *EPSP* excitatory postsynaptic potential, *Fmr1* fragile X mental retardation 1, *GABAR* GABA receptor, *GAD* glutamate decarboxylase, *HIP* hippocampus, *IPSP* inhibitory postsynaptic potential, *STR* striatum

## Conclusions and prospects

**Fig. 1:**
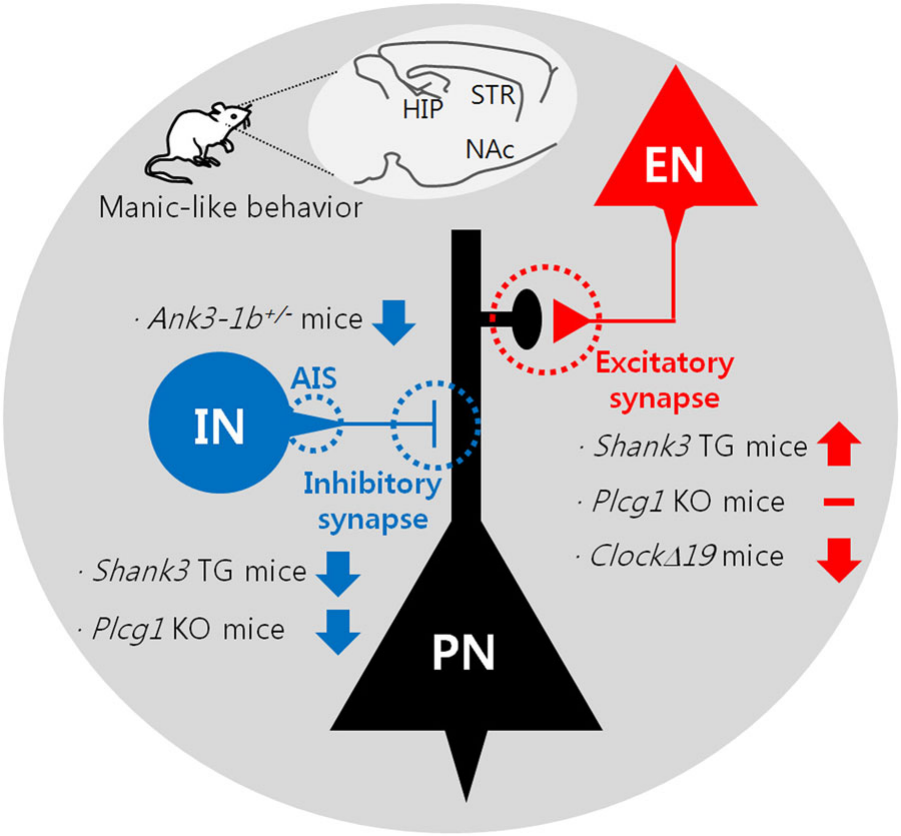
Summary of the E/I synaptic dysfunction in animal models of mania. In the hippocampus of *Shank3* TG mice, excitatory synaptic function is increased, while inhibitory synaptic function is decreased. In the hippocampus and striatum of forebrain-specific *Plcg1* KO mice, excitatory synaptic function is normal, but inhibitory synaptic function is decreased. In the NAc of *ClockΔ19* mutant mice, excitatory synaptic function is decreased. In the brain regions of *Ank3-1b*^+/*−*^ mice, function of the AIS of the PV-positive interneuron is impaired. EN excitatory neuron, HIP hippocampus, IN inhibitory neuron, PN principal neuron, STR striatum

As we summarized in this review, there is an increasing number of evidence supporting the pathogenic role of E/I synaptic dysfunction in mania (Fig. 1). Nevertheless, there are many limitations and questions remaining from animal model studies. First, brain regions, neural circuits, and neuronal cell types mediating specific behavioral phenotypes of mania need to be further dissected. For example, locomotor hyperactivity and hyperhedonic behavior may be mediated by synaptic changes of different brain regions, such as the dorsal striatum and NAc, respectively^35,58^. Recent advances in viral-mediated gene delivery combined with optogenetic tools and brain-clearing technology have revealed the structural and functional anatomy of neural circuits critical for several depression-related behaviors^80,81^. Similar approaches could be applied to the abovementioned animal models of mania to test whether activation or inhibition of specific neural circuits could rescue a subset of behavioral phenotypes. Second, E/I synaptic changes preceding the onset of behavioral phenotypes may be identified and the effect of preventing such earlier synaptic changes could be investigated. In most studies so far, morphological and functional changes of the E/I synapses have been characterized at the ages when the manic-like behaviors of animal models are fully developed. Therefore, it is possible that those E/I synaptic changes may include some compensatory and homeostatic responses of neurons and/or neural circuits, and, thus, could not be causally associated with the behavioral phenotypes. Notably, in the case of schizophrenia, earlier intervention during the juvenile stage has shown to prevent adult onset of behavioral deficits in animal models^82^. Third, E/I synaptic mechanisms underlying the differential responses to certain mood stabilizers could be investigated using animal models. Although lithium still remains the first-line treatment for BD, only about 30% of BD patients show full responses to lithium treatment^83^. Despite the poorly defined neurobiological mechanism, a few genome-wide association studies have linked the variants of genes functioning in the E/I synapses, such as *GRIA2* (for the glutamate receptor, ionotropic, AMPA2) and *GADL1* (for the glutamate decarboxylase-like 1), to the differential responses to lithium treatment^84,85^. In this regard, it is intriguing that the manic-like behaviors of *Shank3* TG mice were selectively rescued by VPA, but not by lithium^16^. More comprehensive analysis of behavioral and synaptic changes in the animal models of mania after single or mixed treatment with various drugs for BD will be an interesting direction of future study. Last but not least, the animal model studies need to be converged with clinical research on patients^86^. Advances in neuroimaging and induced pluripotent stem cell technologies have already narrowed the gaps between the preclinical and clinical studies of BD^87,88^, which, when combined, will provide important insights toward understanding the pathophysiology and potential treatment of this complex and heterogeneous mental illness.

## References

[1] Grande I, Berk M, Birmaher B, Vieta E (2016). Bipolar disorder. Lancet.

[2] Merikangas KR (2011). Prevalence and correlates of bipolar spectrum disorder in the world mental health survey initiative. Arch. Gen. Psychiatry.

[3] Logan RW, McClung CA (2016). Animal models of bipolar mania: the past, present and future. Neuroscience.

[4] Nestler EJ, Hyman SE (2010). Animal models of neuropsychiatric disorders. Nat. Neurosci..

[5] Joyce PR (1995). Urinary catecholamines and plasma hormones predict mood state in rapid cycling bipolar affective disorder. J. Affect. Disord..

[6] Geddes JR, Miklowitz DJ (2013). Treatment of bipolar disorder. Lancet.

[7] Nelson SB, Valakh V (2015). Excitatory/Inhibitory balance and circuit homeostasis in autism spectrum disorders. Neuron.

[8] Lee E, Lee J, Kim E (2017). Excitation/inhibition imbalance in animal models of autism spectrum disorders. Biol. Psychiatry.

[9] Zoghbi HY, Bear MF (2012). Synaptic dysfunction in neurodevelopmental disorders associated with autism and intellectual disabilities. Cold Spring Harb. Perspect. Biol..

[10] Kehrer C, Maziashvili N, Dugladze T, Gloveli T (2008). Altered excitatory-inhibitory balance in the NMDA-hypofunction model of schizophrenia. Front. Mol. Neurosci..

[11] Sudhof TC (2008). Neuroligins and neurexins link synaptic function to cognitive disease. Nature.

[12] Nakazawa K (2012). GABAergic interneuron origin of schizophrenia pathophysiology. Neuropharmacology.

[13] Benes FM, Berretta S (2001). GABAergic interneurons: implications for understanding schizophrenia and bipolar disorder. Neuropsychopharmacology.

[14] Kato T, Kasahara T, Kubota-Sakashita M, Kato TM, Nakajima K (2016). Animal models of recurrent or bipolar depression. Neuroscience.

[15] Sheng M, Kim E (2000). The Shank family of scaffold proteins. J. Cell Sci..

[16] Han K (2013). SHANK3 overexpression causes manic-like behaviour with unique pharmacogenetic properties. Nature.

[17] Lee Y (2017). Integrative analysis of brain region-specific Shank3 interactomes for understanding the heterogeneity of neuronal pathophysiology related to SHANK3 mutations. Front. Mol. Neurosci..

[18] Jiang YH, Ehlers MD (2013). Modeling autism by SHANK gene mutations in mice. Neuron.

[19] Monteiro P, Feng G (2017). SHANK proteins: roles at the synapse and in autism spectrum disorder. Nat. Rev. Neurosci..

[20] Choi SY, Han K (2015). Emerging role of synaptic actin-regulatory pathway in the pathophysiology of mood disorders. Anim. Cells Syst. (Seoul).

[21] Lee B (2017). Age-dependent decrease of GAD65/67 mRNAs but normal densities of GABAergic interneurons in the brain regions of Shank3-overexpressing manic mouse model. Neurosci. Lett..

[22] Peca J (2011). Shank3 mutant mice display autistic-like behaviours and striatal dysfunction. Nature.

[23] Lee J (2015). Shank3-mutant mice lacking exon 9 show altered excitation/inhibition balance, enhanced rearing, and spatial memory deficit. Front. Cell. Neurosci..

[24] Zhou Y (2016). Mice with Shank3 mutations associated with ASD and schizophrenia display both shared and distinct defects. Neuron.

[25] Lee Y (2017). Striatal transcriptome and interactome analysis of Shank3-overexpressing mice reveals the connectivity between Shank3 and mTORC1 Signaling. Front. Mol. Neurosci..

[26] Chiu CT, Wang Z, Hunsberger JG, Chuang DM (2013). Therapeutic potential of mood stabilizers lithium and valproic acid: beyond bipolar disorder. Pharmacol. Rev..

[27] Kim MJ, Kim E, Ryu SH, Suh PG (2000). The mechanism of phospholipase C-gamma1 regulation. Exp. Mol. Med..

[28] Cunha C, Brambilla R, Thomas KL (2010). A simple role for BDNF in learning and memory?. Front. Mol. Neurosci..

[29] Park H, Poo MM (2013). Neurotrophin regulation of neural circuit development and function. Nat. Rev. Neurosci..

[30] Turecki G (1998). Evidence for a role of phospholipase C-gamma1 in the pathogenesis of bipolar disorder. Mol. Psychiatry.

[31] Lovlie R, Berle JO, Stordal E, Steen VM (2001). The phospholipase C-gamma1 gene (PLCG1) and lithium-responsive bipolar disorder: re-examination of an intronic dinucleotide repeat polymorphism. Psychiatr. Genet..

[32] Yang YR (2017). Forebrain-specific ablation of phospholipase Cgamma1 causes manic-like behavior. Mol. Psychiatry.

[33] Geddes JR, Burgess S, Hawton K, Jamison K, Goodwin GM (2004). Long-term lithium therapy for bipolar disorder: systematic review and meta-analysis of randomized controlled trials. Am. J. Psychiatry.

[34] Calabresi P, Picconi B, Tozzi A, Ghiglieri V, Di Filippo M (2014). Direct and indirect pathways of basal ganglia: a critical reappraisal. Nat. Neurosci..

[35] Kravitz AV (2010). Regulation of parkinsonian motor behaviours by optogenetic control of basal ganglia circuitry. Nature.

[36] Bennett V, Healy J (2009). Membrane domains based on ankyrin and spectrin associated with cell-cell interactions. Cold Spring Harb. Perspect. Biol..

[37] Nelson AD, Jenkins PM (2017). Axonal membranes and their domains: assembly and function of the axon initial segment and node of Ranvier. Front. Cell. Neurosci..

[38] Ferreira MA (2008). Collaborative genome-wide association analysis supports a role for ANK3 and CACNA1C in bipolar disorder. Nat. Genet..

[39] Psychiatric GWAS Consortium Bipolar Disorder Working Group. (2011). Large-scale genome-wide association analysis of bipolar disorder identifies a new susceptibility locus near ODZ4. Nat. Genet..

[40] Muhleisen TW (2014). Genome-wide association study reveals two new risk loci for bipolar disorder. Nat. Commun..

[41] Leussis MP (2013). The ANK3 bipolar disorder gene regulates psychiatric-related behaviors that are modulated by lithium and stress. Biol. Psychiatry.

[42] Zhou D (1998). AnkyrinG is required for clustering of voltage-gated Na channels at axon initial segments and for normal action potential firing. J. Cell Biol..

[43] Rueckert EH (2013). Cis-acting regulation of brain-specific ANK3 gene expression by a genetic variant associated with bipolar disorder. Mol. Psychiatry.

[44] Lopez AY (2017). Ankyrin-G isoform imbalance and interneuronopathy link epilepsy and bipolar disorder. Mol. Psychiatry.

[45] Zhu S (2017). Genetic disruption of ankyrin-G in adult mouse forebrain causes cortical synapse alteration and behavior reminiscent of bipolar disorder. Proc. Natl Acad. Sci. USA.

[46] Smith KR (2014). Psychiatric risk factor ANK3/ankyrin-G nanodomains regulate the structure and function of glutamatergic synapses. Neuron.

[47] McClung CA (2007). Circadian genes, rhythms and the biology of mood disorders. Pharmacol. Ther..

[48] McClung CA (2013). How might circadian rhythms control mood? Let me count the ways. Biol. Psychiatry.

[49] Mohawk JA, Green CB, Takahashi JS (2012). Central and peripheral circadian clocks in mammals. Annu. Rev. Neurosci..

[50] Benedetti F (2003). Influence of CLOCK gene polymorphism on circadian mood fluctuation and illness recurrence in bipolar depression. Am. J. Med. Genet. B Neuropsychiatr. Genet..

[51] Nievergelt CM (2006). Suggestive evidence for association of the circadian genes PERIOD3 and ARNTL with bipolar disorder. Am. J. Med. Genet. B Neuropsychiatr. Genet..

[52] King DP (1997). Positional cloning of the mouse circadian clock gene. Cell.

[53] Antoch MP (1997). Functional identification of the mouse circadian Clock gene by transgenic BAC rescue. Cell.

[54] Roybal K (2007). Mania-like behavior induced by disruption of CLOCK. Proc. Natl Acad. Sci. USA.

[55] McClung CA (2005). Regulation of dopaminergic transmission and cocaine reward by the Clock gene. Proc. Natl Acad. Sci. USA.

[56] Ozburn AR, Larson EB, Self DW, McClung CA (2012). Cocaine self-administration behaviors in ClockDelta19 mice. Psychopharmacology (Berl.)..

[57] Sidor MM (2015). Daytime spikes in dopaminergic activity drive rapid mood-cycling in mice. Mol. Psychiatry.

[58] Russo SJ, Nestler EJ (2013). The brain reward circuitry in mood disorders. Nat. Rev. Neurosci..

[59] Dzirasa K (2010). Lithium ameliorates nucleus accumbens phase-signaling dysfunction in a genetic mouse model of mania. J. Neurosci..

[60] Parekh, P. K. et al. Altered GluA1 function and accumbal synaptic plasticity in the ClockΔ19 model of bipolar mania. *Biol Psychiatry* 2017; pii: S0006-3223(17)31721-3. 10.1016/j.biopsych.2017.06.022 PMC5745309 28780133

[61] Spencer S (2012). A mutation in CLOCK leads to altered dopamine receptor function. J. Neurochem..

[62] Harvey AG (2011). Sleep and circadian functioning: critical mechanisms in the mood disorders?. Annu. Rev. Clin. Psychol..

[63] Gessa GL, Pani L, Fadda P, Fratta W (1995). Sleep deprivation in the rat: an animal model of mania. Eur. Neuropsychopharmacol..

[64] Valvassori SS (2017). Lithium ameliorates sleep deprivation-induced mania-like behavior, hypothalamic-pituitary-adrenal (HPA) axis alterations, oxidative stress and elevations of cytokine concentrations in the brain and serum of mice. Bipolar Disord..

[65] Szabo ST (2009). Glutamate receptors as targets of protein kinase C in the pathophysiology and treatment of animal models of mania. Neuropharmacology.

[66] Kanazawa LK (2016). Quercetin reduces manic-like behavior and brain oxidative stress induced by paradoxical sleep deprivation in mice. Free Radic. Biol. Med..

[67] Chen BS, Roche KW (2007). Regulation of NMDA receptors by phosphorylation. Neuropharmacology.

[68] Jenkins MA (2014). Regulation of GluA1 alpha-amino-3-hydroxy-5-methyl-4-isoxazolepropionic acid receptor function by protein kinase C at serine-818 and threonine-840. Mol. Pharmacol..

[69] McDermott CM (2003). Sleep deprivation causes behavioral, synaptic, and membrane excitability alterations in hippocampal neurons. J. Neurosci..

[70] Vecsey CG (2009). Sleep deprivation impairs cAMP signalling in the hippocampus. Nature.

[71] Malenka RC, Bear MF (2004). LTP and LTD: an embarrassment of riches. Neuron.

[72] Chen C, Hardy M, Zhang J, LaHoste GJ, Bazan NG (2006). Altered NMDA receptor trafficking contributes to sleep deprivation-induced hippocampal synaptic and cognitive impairments. Biochem. Biophys. Res. Commun..

[73] McDermott CM, Hardy MN, Bazan NG, Magee JC (2006). Sleep deprivation-induced alterations in excitatory synaptic transmission in the CA1 region of the rat hippocampus. J. Physiol..

[74] Liu ZW, Faraguna U, Cirelli C, Tononi G, Gao XB (2010). Direct evidence for wake-related increases and sleep-related decreases in synaptic strength in rodent cortex. J. Neurosci..

[75] Winters BD, Huang YH, Dong Y, Krueger JM (2011). Sleep loss alters synaptic and intrinsic neuronal properties in mouse prefrontal cortex. Brain Res..

[76] Miyamoto S, Duncan GE, Marx CE, Lieberman JA (2005). Treatments for schizophrenia: a critical review of pharmacology and mechanisms of action of antipsychotic drugs. Mol. Psychiatry.

[77] Rapoport SI, Basselin M, Kim HW, Rao JS (2009). Bipolar disorder and mechanisms of action of mood stabilizers. Brain Res. Rev..

[78] Schloesser RJ, Martinowich K, Manji HK (2012). Mood-stabilizing drugs: mechanisms of action. Trends Neurosci..

[79] Quiroz JA, Gould TD, Manji HK (2004). Molecular effects of lithium. Mol. Interv..

[80] Lammel S (2012). Input-specific control of reward and aversion in the ventral tegmental area. Nature.

[81] Lammel S, Tye KM, Warden MR (2014). Progress in understanding mood disorders: optogenetic dissection of neural circuits. Genes Brain Behav..

[82] Marin O (2016). Developmental timing and critical windows for the treatment of psychiatric disorders. Nat. Med..

[83] Alda M (2015). Lithium in the treatment of bipolar disorder: pharmacology and pharmacogenetics. Mol. Psychiatry.

[84] Perlis RH (2009). A genomewide association study of response to lithium for prevention of recurrence in bipolar disorder. Am. J. Psychiatry.

[85] Chen CH (2014). Variant GADL1 and response to lithium therapy in bipolar I disorder. N. Engl. J. Med..

[86] Kaiser T, Feng G (2015). Modeling psychiatric disorders for developing effective treatments. Nat. Med..

[87] Maletic V, Raison C (2014). Integrated neurobiology of bipolar disorder. Front. Psychiatry.

[88] Mertens J (2015). Differential responses to lithium in hyperexcitable neurons from patients with bipolar disorder. Nature.

[89] Farooq M (2017). Lithium increases synaptic GluA2 in hippocampal neurons by elevating the delta-catenin protein. Neuropharmacology.

[90] Tyagarajan SK (2011). Regulation of GABAergic synapse formation and plasticity by GSK3beta-dependent phosphorylation of gephyrin. Proc. Natl Acad. Sci. USA.

[91] Motohashi N, Ikawa K, Kariya T (1989). GABAB receptors are up-regulated by chronic treatment with lithium or carbamazepine. GABA hypothesis of affective disorders?. Eur. J. Pharmacol..

[92] de Bartolomeis A, Tomasetti C, Cicale M, Yuan PX, Manji HK (2012). Chronic treatment with lithium or valproate modulates the expression of Homer1b/c and its related genes Shank and Inositol 1,4,5-trisphosphate receptor. Eur. Neuropsychopharmacol..

[93] Gideons ES, Lin PY, Mahgoub M, Kavalali ET, Monteggia LM (2017). Chronic lithium treatment elicits its antimanic effects via BDNF-TrkB dependent synaptic downscaling. Elife.

[94] Ma J, Zhang GY (2003). Lithium reduced N-methyl-D-aspartate receptor subunit 2A tyrosine phosphorylation and its interactions with Src and Fyn mediated by PSD-95 in rat hippocampus following cerebral ischemia. Neurosci. Lett..

[95] Hashimoto R, Hough C, Nakazawa T, Yamamoto T, Chuang DM (2002). Lithium protection against glutamate excitotoxicity in rat cerebral cortical neurons: involvement of NMDA receptor inhibition possibly by decreasing NR2B tyrosine phosphorylation. J. Neurochem..

[96] Du J (2004). Modulation of synaptic plasticity by antimanic agents: the role of AMPA glutamate receptor subunit 1 synaptic expression. J. Neurosci..

[97] Du J (2008). The role of hippocampal GluR1 and GluR2 receptors in manic-like behavior. J. Neurosci..

[98] Du J (2007). The anticonvulsants lamotrigine, riluzole, and valproate differentially regulate AMPA receptor membrane localization: relationship to clinical effects in mood disorders. Neuropsychopharmacology.

[99] Kim HJ, Thayer SA (2009). Lithium increases synapse formation between hippocampal neurons by depleting phosphoinositides. Mol. Pharmacol..

[100] Martin PM (2018). DIXDC1 contributes to psychiatric susceptibility by regulating dendritic spine and glutamatergic synapse density via GSK3 and Wnt/beta-catenin signaling. Mol. Psychiatry.

[101] Liu ZH, Chuang DM, Smith CB (2011). Lithium ameliorates phenotypic deficits in a mouse model of fragile X syndrome. Int. J. Neuropsychopharmacol..

[102] Gebhardt C, Cull-Candy SG (2010). Lithium acts as a potentiator of AMPAR currents in hippocampal CA1 cells by selectively increasing channel open probability. J. Physiol..

[103] Shim SS, Hammonds MD, Ganocy SJ, Calabrese JR (2007). Effects of sub-chronic lithium treatment on synaptic plasticity in the dentate gyrus of rat hippocampal slices. Prog. Neuropsychopharmacol. Biol. Psychiatry.

[104] Colino A, Garcia-Seoane JJ, Valentin A (1998). Action potential broadening induced by lithium may cause a presynaptic enhancement of excitatory synaptic transmission in neonatal rat hippocampus. Eur. J. Neurosci..

[105] Valentin A, Garcia-Seoane JJ, Colino A (1997). Lithium enhances synaptic transmission in neonatal rat hippocampus. Neuroscience.

[106] Peineau S (2007). LTP inhibits LTD in the hippocampus via regulation of GSK3beta. Neuron.

[107] Nau H, Loscher W (1982). Valproic acid: brain and plasma levels of the drug and its metabolites, anticonvulsant effects and gamma-aminobutyric acid (GABA) metabolism in the mouse. J. Pharmacol. Exp. Ther..

[108] Johannessen CU, Petersen D, Fonnum F, Hassel B (2001). The acute effect of valproate on cerebral energy metabolism in mice. Epilepsy Res..

[109] Hassel B, Iversen EG, Gjerstad L, Tauboll E (2001). Up-regulation of hippocampal glutamate transport during chronic treatment with sodium valproate. J. Neurochem..

[110] Takuma K (2014). Chronic treatment with valproic acid or sodium butyrate attenuates novel object recognition deficits and hippocampal dendritic spine loss in a mouse model of autism. Pharmacol. Biochem. Behav..

[111] Baldino F, Geller HM (1981). Sodium valproate enhancement of gamma-aminobutyric acid (GABA) inhibition: electrophysiological evidence for anticonvulsant activity. J. Pharmacol. Exp. Ther..

[112] Gean PW, Huang CC, Hung CR, Tsai JJ (1994). Valproic acid suppresses the synaptic response mediated by the NMDA receptors in rat amygdalar slices. Brain Res. Bull..

[113] Ko GY, Brown-Croyts LM, Teyler TJ (1997). The effects of anticonvulsant drugs on NMDA-EPSP, AMPA-EPSP, and GABA-IPSP in the rat hippocampus. Brain Res. Bull..

[114] Martin ED, Pozo MA (2004). Valproate reduced excitatory postsynaptic currents in hippocampal CA1 pyramidal neurons. Neuropharmacology.

[115] Hassel B, Tauboll E, Gjerstad L (2001). Chronic lamotrigine treatment increases rat hippocampal GABA shunt activity and elevates cerebral taurine levels. Epilepsy Res..

[116] Sitges M, Chiu LM, Guarneros A, Nekrassov V (2007). Effects of carbamazepine, phenytoin, lamotrigine, oxcarbazepine, topiramate and vinpocetine on Na+ channel-mediated release of [3H]glutamate in hippocampal nerve endings. Neuropharmacology.

[117] Sitges M, Guarneros A, Nekrassov V (2007). Effects of carbamazepine, phenytoin, valproic acid, oxcarbazepine, lamotrigine, topiramate and vinpocetine on the presynaptic Ca2+ channel-mediated release of [3H]glutamate: comparison with the Na+ channel-mediated release. Neuropharmacology.

[118] Wang SJ, Huang CC, Hsu KS, Tsai JJ, Gean PW (1996). Presynaptic inhibition of excitatory neurotransmission by lamotrigine in the rat amygdalar neurons. Synapse.

[119] Lee CY, Fu WM, Chen CC, Su MJ, Liou HH (2008). Lamotrigine inhibits postsynaptic AMPA receptor and glutamate release in the dentate gyrus. Epilepsia.

[120] Cunningham MO, Jones RS (2000). The anticonvulsant, lamotrigine decreases spontaneous glutamate release but increases spontaneous GABA release in the rat entorhinal cortex in vitro. Neuropharmacology.

[121] Braga MF, Aroniadou-Anderjaska V, Post RM, Li H (2002). Lamotrigine reduces spontaneous and evoked GABAA receptor-mediated synaptic transmission in the basolateral amygdala: implications for its effects in seizure and affective disorders. Neuropharmacology.

[122] Kamal SM (2010). Pharmacological modulation of brain levels of glutamate and GABA in rats exposed to total sleep deprivation. J. Exp. Pharmacol..

[123] Booker SA, Pires N, Cobb S, Soares-da-Silva P, Vida I (2015). Carbamazepine and oxcarbazepine, but not eslicarbazepine, enhance excitatory synaptic transmission onto hippocampal CA1 pyramidal cells through an antagonist action at adenosine A1 receptors. Neuropharmacology.

[124] Granger P (1995). Modulation of the gamma-aminobutyric acid type A receptor by the antiepileptic drugs carbamazepine and phenytoin. Mol. Pharmacol..

[125] Ambrosio AF (2001). Inhibition of glutamate release by BIA 2-093 and BIA 2-024, two novel derivatives of carbamazepine, due to blockade of sodium but not calcium channels. Biochem. Pharmacol..

[126] Giustizieri M (2008). Differential effect of carbamazepine and oxcarbazepine on excitatory synaptic transmission in rat hippocampus. Synapse.

